# Prevalence of Genetic Determinants and Phenotypic Resistance to Ciprofloxacin in *Campylobacter jejuni* from Lithuania

**DOI:** 10.3389/fmicb.2018.00203

**Published:** 2018-02-14

**Authors:** Jurgita Aksomaitiene, Sigita Ramonaite, John E. Olsen, Mindaugas Malakauskas

**Affiliations:** ^1^Department of Food Safety and Quality, Veterinary Academy, Lithuanian University of Health Sciences, Kaunas, Lithuania; ^2^Department of Veterinary and Animal Sciences, University of Copenhagen, Frederiksberg, Denmark

**Keywords:** *Campylobacter jejuni*, antimicrobial resistance, sequencing identification, ciprofloxacin, QRDR

## Abstract

Recently, the number of reports on isolation of ciprofloxacin resistant *Campylobacter jejuni* has increased worldwide. The aim of this study was to determine the prevalence of resistance to ciprofloxacin and its genetic determinants among *C. jejuni* isolated from humans (*n* = 100), poultry products (*n* = 96) and wild birds (*n* = 96) in Lithuania. 91.4% of the *C. jejuni* isolates were phenotypically resistant to ciprofloxacin. DNA sequence analyses of the *gyrA* gene from 292 isolates revealed that a change in amino acid sequence, Thr86Ile, was the main substition conferring resistance to ciprofloxacin. This change was significantly associated with isolates from poultry products (*P* < 0.05) and humans (*P* < 0.05). A total of 26.7% of *C. jejuni* isolates from human (*n* = 47), poultry products (*n* = 30) and wild bird (*n* = 1), had a mutation from Ser at position 22, and six had an additional mutation from Ala at position 39. Eight isolates from poultry and two isolates from human, corresponding to 67.0% of isolates with MICs ≥128 μg/ml, showed missense mutations Thr86Ile (ACA → ATA) and Ser22Gly (AGT → GGT) together, whereas isolates without these mutations showed lower MIC values (from 4 to 64 μg/ml). Two hundred forty-five *C. jejuni* isolates showed one or more silent mutations, and 32.4% of examined isolates possessed six silent mutations. In addition to the ciprofloxacin resistant isolates harboring only Thr86Ile point mutation (110 isolates), the current study identified resistant isolates (*n* = 101) harboring additional point mutations (Ser22Gly, Ala39Ser, Arg48Lys, Thr85Ala Ala122Ser, Glu136Asp, Vall49Ile), and strains (*n* = 57) having only Glu136Asp point mutation. The study highlight the potential public health problem with elevated ciprofloxacin resistance in *Campylobacters* from poultry meat, wild birds and humans, and the need for extensive surveillance enabling to follow changes of antimicrobial resistance development in this species.

## Introduction

*Campylobacter jejuni* is a leading foodborne pathogen in many countries (Engberg et al., 2001). It colonizes the gastrointestinal tracts of a wide range of wild, domestic, and livestock animals, and foods of animal origin are a significant source of campylobacteriosis in humans (Colles et al., 2008; Silva et al., 2011). Transmission of *C. jejuni* to humans most often occurs via to consumption of contaminated food, especially chicken meat, or by direct contact with feces (Miller et al., 2010). Although most infections are mild and self-limiting and do not require antimicrobial therapy, treatment is indicated in immunocompromised patients, or if the infection is extraintestinal (Goodman et al., 1984). Fluoroquinolones, such as ciprofloxacin, are the antimicrobial agents of choice for treatment of campylobacteriosis (Guerrant et al., 2001; Blaser and Engberg, 2008). Other antimicrobials, however, such as erythromycin, azithromycin and gentamicin may be viable alternatives (Wieczorek and Osek, 2013; Wimalarathna et al., 2013). Moreover, fluoroquinolones have been used as first-line antibiotics against bacterial gastroenteritis in the absence of microbiological diagnosis (Wieczorek, 2011). The quinolones target two large, bacterial enzymes, DNA gyrase and topoisomerase IV, and binding to these enzymes inhibit the synthesis of bacterial DNA, causing cell death. Resistance to fluoroquinolones is mainly due to amino acids substitutions in the quinolone resistance-determining region (QRDR) of the topoisomerase (Wieczorek and Osek, 2013). Due to common use of flouroquinolones in livestock in some countries, and for treatment of campylobacteriosis in humans, resistance to this antimicrobial group has emerged worldwide in *C. jejuni* and has become a public health issue (Abay et al., 2014; Sahin et al., 2015). Resistance to quinolones in *Campylobacter* is usually mediated by a single point mutation in the quinolone resistance-determining region (QRDR) of the *gyrA* gene at codon 86 (ACA → ATA), leading to isoleucine substitution for threonine (Wang et al., 1993; Ruiz et al., 1998; Zirnstein et al., 1999). This alteration is most often associated with high MIC values for fluoroquinolones (Wang et al., 1993). Other substitutions in the QRDR are also known and described, but these changes are less common (Wang et al., 1993; Hakanen et al., 2003).

The prevalence of resistance to ciprofloxacin in *Campylobacter* species vary considerably between countries, with high prevalence reported in Poland (Wieczorek and Osek, 2013), Italy (Di Giannatale et al., 2014), Switzerland (Kittl et al., 2013) and some other EU countries (EFSA, 2014a,b). The rapid emergence of antimicrobial resistance is a cause for global concern (Engberg et al., 2001; Hein et al., 2003). To our knowledge, there are no published data on the genetic background for flouroquinolone resistance in *C. jejuni* isolated from different sources in Lithuania. Therefore, the aim of this study was to determine the prevalence of resistance to ciprofloxacin among *C. jejuni* isolated from humans, poultry products and wild birds in Lithuania, and to assess the *gyrA* mutations responsible for the resistance among the *C. jejuni* isolates.

## Materials and methods

### Bacterial strains and culture conditions

A total of 292 *C. jejuni* isolates from infected children (*n* = 100), raw (*n* = 77), and marinated (*n* = 19) broiler products and wild birds: pigeons (*n* = 39) and crows (*n* = 57) were selected. Food and wild bird isolates were from the *Campylobacter* collection at the Department of Food Safety and Quality, Veterinary Academy, Lithuanian University of Health Sciences. The human *C. jejuni* isolates were received from the Microbiological laboratory of Kaunas Clinical Hospital and were isolated in the period from 2011 to 2012. Identification of *Campylobacter* isolates was performed with multiplex PCR as described by Wang et al. (2002) with the minor modifications described previously by Ramonaite et al. (2015).

*Campylobacter* isolates were stored as frozen stocks at −80°C in in brain heart infusion broth (BHI) (Oxoid Ltd., Basingstoke, UK) with 30% glycerol (Stanlab, Poland). They were recovered from frozen stocks on Blood agar base No. 2 (Oxoid, Basingstoke, Hampshire, England) supplemented with 5% defibrinated horse blood (E&O Laboratories, Burnhouse, Bonnybridge, Scotland) and incubated under microaerophilic conditions (5% oxygen, 10% carbon dioxide and 85% nitrogen) at 37°C for 48 h.

### Antimicrobial resistance testing

Minimum inhibitory concentrations (MIC) were determined according to the recommendations of the Clinical and Laboratory Standarts Institute (CLSI) (CLSI, 2006). Antimicrobial susceptibility was evaluated using the quality control strain *C. jejuni* ATCC 33560. Briefly, suspension of *C. jejuni* isolates adjusted to an *OD*_600_ = 0.1 were prepared in phosphate buffered saline and inoculated onto Mueller-Hinton agar (LiofilChem, Milan Italy) supplemented with 5% lysed sheep blood (E&O Laboratories, Burnhouse, Bonnybridge, Scotland) and ciprofloxacin (Sigma-Aldrich, Saint-Louis, USA) in concentrations ranging from 0.25 to 256 μg/ml and incubated under microaerophilic conditions at 42°C for 24 h. The MIC interpretive criterion for resistance to ciprofloxacin was ≥4 μg/ml.

### DNA extraction

Bacterial isolates were grown at 37°C on blood agar plates for 48 h under microaerophilic conditions. After sufficient growth was obtained, one 1 μl loopful of bacteria was suspended in Eppendorf tubes containing 200 μl of PrepMan Ultra Sample Preparation Reagent (PrepMan™ Ultra, Applied Biosystems, USA). DNA extraction was carried out following the instructions of the supplier including heating of bacterial suspension at 100°C for 10 min., centrifugation at 16,000 g for 3 min. and transferring the supernatants into new tubes before storage in the freezer at −20°C until use.

### PCR detection of antibiotic resistance determinants

The QRDR of the *gyrA* gene of the *C. jejuni* isolates was amplified by MAMA PCR as described by Zirstein (Zirnstein et al., 1999) using *GzgyrA5* and *GzgyrA6* primers for amplification of a 673 bp product (Table 1). Forward primer CampyMAMA*gyrA1* and a reverse primer CampyMAMA*gyrA5* (Table 1) were used to generate a 256 bp PCR product that was a positive indication of the presence of the Thr-86-Ile (ACA → ATA) mutation in the *C. jejuni gyrA* gene. Primer *GZgyrA4*, a conserved reverse primer, was used in conjugation with primer CampyMAMA*gyrA1* to produce a positive PCR control product of 368 bp with any *C. jejuni gyrA* gene. PCR cycling conditions were as follow: 3 min initial denaturation at 94°C, followed by 30 cycles of denaturation at 94°C for 1 min, annealing at 54°C for 1 min and extension at 72°C for 1 min, with a final step at 72°C for 5 min.

**Table 1:** Primers used for PCR in this study.

	**MAMA PCR**		
**Primer**	**Sequence (5′ → 3′)**	**Direction**	**References**
*GZgyrA4*	cagtataacgcatcgcagcg	Reverse	Zirnstein et al., 1999
GZ*gyrA5*	atttttagcaaagattctgat	Forward	
GZ*gyrA6*	ccataaattattccacctgt	Reverse	
GZ*gyrA7*	ttattataggtcgtgctttg	Nested forward	
GZ*gyrA8*	tagaaggtaaaacatcaggtt	Nested reverse	
CampyMAMA*gyrA*1	tttttagcaaagattctgat	Forward	
CampyMAMA*gyrA*5	caaagcatcataaactgcaa	Reverse	
*gyrA*	gctgatgcaaaagkttaatatgc	Forward	Ragimbeau et al., 2014
*gyrA*	tttgtcgccatacctacagc	Reverse	

### DNA sequencing and sequence analysis

Nested primers *GZgyrA7* and *GZgyrA8* (Table 1), which are internal to the 673 bp *gyrA* PCR products produced above, were used for sequencing. The partial gene sequence of *gyrA*, generated by use of CampyMAMA*gyrA1* and a reverse primer CampyMAMA*gyrA5* targeting the quinolone resistance determining region (QRDR), was sequenced with the forward and reverse primers described by Ragimbeau et al. (Table 1). The amplification protocol consisted of 95°C for 10 min, followed by 35 cycles of 95°C 30 s, 55°C 30 s, and 72°C 50 s. The reaction was completed by a final extension of 5 min at 72°C.

The PCR amplicons were purified using the GeneJet PCR purification system (Thermo Scientific, EU). Sequencing reactions were carried out using the BigDye Terminator 3.1 cycle Sequencing Kit (Applied Biosystems, USA) according to instructions from the manufacturer. Duplicate forward and reverse sequencing reactions were run with forward and reverse primers, respectively, for each sample. The samples were analyzed with a 3500 Genetic Analyzer (Applied Biosystems). The BioNumerics program 7.0 (Applied Maths NV, USA, EU) was used to evaluate the specific genomic mutations associated with resistance to ciprofloxacin, including comparison to the reference *C. jejuni* strain ATCC 700819 genome (NCTC11168).

### Statistical analysis

The statistical package SPSS (Statistics 20, IBM) was used. Comparison of association between phenotypic resistance and resistance genes in *C. jejuni* from humans, poultry products and wild birds isolates and distributions of resistant isolates were evaluated using the Chi-square test and Fisher's exact test. A *p*-value of < 0.05 was used to indicate statistically significant results.

## Results

### Phenotypic antimicrobial resistance

Among the 292 isolates tested, 267 (91.4%) were phenotypically resistant to ciprofloxacin (Table 2). In particular, all *C. jejuni* isolates from poultry products were resistant to ciprofloxacin with MIC ranging from 4 μg/ml up to 256 μg/ml.

**Table 2:** Antimicrobial resistance of *C. jejuni* from poultry products, humans, and wild-bird samples.

**Source**	**Range tested (μg/ml)**	**MIC of ciprofloxacin (μg/ml)**	**Resistance breakpoint (μg/ml)**	**No. (%) of resistant strains**
Poultry products	0.5–256	4–256	≥4	96 (100)
Humans	0.5–256	1–128	≥4	88 (88.0)
Wild birds	0.5–256	2–64	≥4	83 (86.4)

### PCR typing of resistant isolates

Phenotypic resistance to ciprofloxacin matched genotypic resistance of all isolates from broiler products and the vast majority (93%) of isolates from humans. Out of the 267 ciprofloxacin-resistant isolates, the majority (76.4%; *n* = 204) were positive for the Thr86Ile substitution of the *gyrA* gene as demonstrated by PCR. The Thr86Ile substitution was not detected in 62 wild bird isolates, which showed phenotypic resistance to ciprofloxacin.

### Amino acid sequences of the *gyrA* gene in ciprofloxacin resistant *C. jejuni*

We identified eight different missense amino acid substitutions in the *gyr*A gene of the ciprofloxacin resistant *C. jejuni* isolates (Table S1 in Supplementary Materials). One hundred and ten *C. jejuni* isolates harbored a single Thr86Ile substitution in *gyrA* without any other amino acid changes in this region. One of the other substitutions was Ser22Gly, which is known to confer fluoroquinolones resistance, often along with the Thr86Ile substitution (Jesse et al., 2006). The combined Thr86Ile (ACA → ATA) and Ser22Gly (AGT → GGT) changes were detected in 10 out of the 15 ciprofloxacin-resistant *C. jejuni* isolates with MICs ≥128 μg/ml (67.0%). Seven isolates carried the Ala39Ser substitution, and these isolates also had the Thr86Ile amino acid change. Six isolates carried the Ser22Gly, Ala39Ser and the Thr86Ile changes in combination, whereas only one isolate carried four substitutions: Ser22Gly, Arg48Lys, Thr86Ile, and Glu136Asp. Interestingly, four ciprofloxacin resistant *C. jejuni* isolates had no point mutations in QRDR. Seventy-four ciprofloxacin resistant *C. jejuni* isolates had a Glu136 point mutation. This mutation was significantly more common in isolates from wild birds (*P* < 0.05) than in isolates from poultry product and humans. Table S1 in Supplementary Materials lists the observed combinations of mutations in the *gyrA* QRDR.

The study revealed at least one silent mutation in 245 *C. jejuni* isolates (Table 3), and 6 *C. jejuni* isolates harbored more than six silent mutations. Details on the observed silent mutations in relation to clonal lines can be seen from Table S1 in the Supplementary Material.

**Table 3:** Silent mutations in the *gyrA* QRDR of *C. jejuni* isolates and associated ranges of MICs of ciprofloxacin.

**Number of strains**	**MIC (μg/ml)**	**Nucleotide change**	**Amino acid position**
5	4–256	AAA → AAG	Lys21 → Lys
95	2–256	TAT → TAC	Tyr24 → tyr
3	4	TCT → TCC	Ser28 → Ser
1	16	GAC → GAT	Asp39 → Asp
2	2–32	GAG → GAA	Glu59 → Glu
2	4–8	GAA → GAG	Glu63 → Glu
1	2	CAG → CAA	Gln69 → Gln
7	2–128	GGT → GCC	Gly78 → Gly
141	2–256	CAC → CAT	His81 → His
1	16	AAG → AAA	Lys97 → Lys
3	2–32	GGC → GGT	Gly110 → Gly
2	4–8	GTG → GTC	Val117 → Val
3	4	ATA → ATC	Ile118 → Ile
216	2–256	AGT → AGC	Ser119 → Ser
150	2–128	GCC → GCT	Ala120 → Ala
1	16	AGC → AGT	Ser137 → Ser
2	4–8	AGC → AGT	Ser145 → Ser
1	8	TTC → TTT	Phe149 → Phe
6	4–256	TAT → TAC	Tyr152 → Tyr
181	2–256	AGC → AGT	Ser157 → Ser
127	2–256	GTT → GTC	Val161 → Val

### Association between MLST genotypes and phenotypic antimicrobial resistance

MLST types of the isolates investigated were published by Ramonaite et al. (2015, 2017). In the current investigation, we related QRDR sequence types to MLST type. The *gyrA* gene point-mutations A64G, G118T and C257T were observed in four isolates with ST-257 (CC257), in one isolate with ST-51 (CC443) and one isolate with ST-6413 (CC353). In total 40 isolates having the Thr-86-Ile substitution were assigned to CC353 and 31 of 33 isolates belonging to CC21. More than half of the isolates (57.7%) belonging to an undefined clonal complex is isolated from wild birds and had the Glu136Asp novel mutation (Table S1 in Supplementary Materials).

## Discussion

Ciprofloxacin resistant *Campylobacter* isolates were observed already from the late 1980s, indicating animals as the main source of resistant bacteria. Currently, flouroquinolone resistance in *C. jejuni* is emerging globally and is considered a problem of public health importance (Wardak et al., 2007; Luangtongkum et al., 2009; Wieczorek, 2011). In this study, *C. jejuni* isolates originating from humans, poultry products and wild birds in Lithuania were examined for resistance to ciprofloxacin.

A high prevalence of *C. jejuni* resistance to ciprofloxacin was observed irrespective of the source of isolation (91.4%). This is much higher resistance prevalence than has been reported in other parts of the Baltic region. For example, Estonia reported only 16.7% prevalence of this resistance (Mäesaar et al., 2016). On the other hand, it is very similar to what has previously been reported from isolates obtained from broiler meat in Latvia and Lithuania (87.5 and 84.8%, respectively) (Mäesaar et al., 2016). The Baltic region is not exceptional, as elevated levels of ciprofloxacin resistance in *C. jejuni* have also been reported from Poland (Wieczorek and Osek, 2013), Italy (Di Giannatale et al., 2014), Switzerland (Kittl et al., 2013) and some other EU countries (EFSA, 2014a,b). However, it should be mentioned, that prevalence of resistance can change dramatically over time.

Resistance to fluoroquinolones in *Campylobacter* is mainly due to amino acids substitutions in the quinolone resistance-determining region (QRDR) of *gyrA* (Wieczorek and Osek, 2013). The most frequently observed mutation is the C257T mutation in the *gyrA* gene, which leads to the Thr86Ile substitution in the gyrase, and confers high-level resistance to this class of antimicrobials (Wang et al., 1993; Payot et al., 2006). In confirmation of this and other studies (Kinana et al., 2006; Tang et al., 2017), the Thr86Ile substitution was the most frequently observed amino acid change in the current study. Previous studies (Sonnevend et al., 2006; Boonmar et al., 2007; Wieczorek, 2011; Duarte et al., 2014) have reported that all ciprofloxacin resistant *C. jejuni* carried the Thr86Ile amino acid substitution in the QRDR of *gyrA*. However, while our study showed this mutation in 100% of the broiler products isolates and 98.9% of the human isolates, only 25.3% of the ciprofloxacin-resistant wild bird isolates carried the corresponding mutation. Other mechanisms of resistance, including decreased outer membrane permeability and efflux systems, have been described (Charvalos et al., 1996), and these may contribute to the phenotypic resistance observed in the strains, where no amino acid changes in *gyrA* were observed.

The Ser22Gly substitution alone may not confer ciprofloxacin resistance, since it has been identified in susceptible strains (Jesse et al., 2006; Tang et al., 2017). However, the double substitution, Thr86Ile/Ser22Gly, is known to confer high-level ciprofloxacin resistance in *Campylobacter* (Oishi et al., 2015). We also identified the mutation leading to Ser22Gly substitution in combination with the mutation leading to Thr86Ile substitution, and the presence of both mutations was associated with high MIC values. Interestingly, one of the isolates with this combination carried an additional double novel mutation, from Arg at position 48 and from Glu at position 136. More research is needed to clarified whether these novel mutations contribute to phenotypic ciprofloxacin resistance in *C. jejuni*.

Silent mutation are often described in ciprofloxacin resistant and sensitive strains and a high number of combinations of transitions and mutations may exist (Frasao et al., 2015). We confirmed this observation, and some of the most frequently observed silent mutations at His-81 → His, Ala-119 → Ala, and Ser-120 → Ser corresponds to mutations described in *Campylobacter* isolated in Finland and Brazil (Hakanen et al., 2002; Frasao et al., 2015). Other transitions without amino acid changes were frequently found in the QRDR, generating many different *gyrA* alleles, TCT → TCC at the codon 84 described by Carattoli et al. (2002) and Gly-110 → Gly (GGC → GGT) described by Beckmann et al. (2004). However, we did not observe silent mutation at Ser-75 → Ser (GCT → GAT) and Ser-79 → Ser (CGT → AGT), as described by Frasao et al. (2015).

## Conclusions

In conclusion, the phenotypic and genotypic antimicrobial resistance of *C. jejuni* from human, poultry products and wild birds to ciprofloxacin have been investigated and reported for the first time in Lithuania. Our study demonstrated a high prevalence of ciprofloxacin resistance in the isolates. The data presented here confirmed previous findings that mutations in the *gyrA* gene at position 257 are mainly responsible for ciprofloxacin resistance of *C. jejuni* strains from poultry products and humans. In addition, our results indicated that Glu136Asp mutation is associated with high-level ciprofloxacin resistance among wild bird isolates.

## Author contributions

JA, SR, and MM: conceived of the presented idea; JA and SR: performed laboratory work and analysis; JA: performed statistical analysis, and drafted the manuscript; JO and MM: contributed to the interpretation of the results. All authors provided critical feedback and helped shape the research, analysis and manuscript.

### Conflict of interest statement

The authors declare that the research was conducted in the absence of any commercial or financial relationships that could be construed as a potential conflict of interest.
