# Supplementary material for: Prevalence of Genetic Determinants and Phenotypic Resistance to Ciprofloxacin in Campylobacter jejuni from Lithuania
Source: Front Microbiol. 2018 Feb 14;9:203. doi: 10.3389/fmicb.2018.00203 (PMC5817067; doi:10.3389/fmicb.2018.00203)
Supplement: Supplementary file 1 [file Table1.docx]

Supplementary Material

**Prevalence of genetic determinants and phenotypic resistance to ciprofloxacin in *Campylobacter jejuni* from Lithuania**

**Jurgita Aksomaitiene^1*^, Sigita Ramonaite^1^, John Elmerdahl Olsen^2^, Mindaugas Malakauskas^1^**

^1^Department of Food Safety and Quality, Veterinary Academy, Lithuanian University of Health Sciences, Tilžes 18, Kaunas, Lithuania

^2^Department of Veterinary and Animal Sciences University of Copenhagen, University of Copenhagen, Stigbøjlen 4, Frederiksberg C Denmark

*** Correspondence:**Jurgita Aksomaitiene: [jurgita.aksomaitiene@lsmuni.lt](mailto:jurgita.aksomaitiene@lsmuni.lt)

# Supplementary Figures and Tables

# Supplementary Material

**Table S1 Nucleotide and amino acid changes due to missense mutation in the quinolone resistance-determining region of *gyrA***

**gene of DNA gyrase of 292 isolates of *C. jejuni***

| **CC (ST)** | **Number of strains** | **MIC (µg/ml)** | Nucleic acid codons and corresponding amino acid changes in QRDR of *gyrA* gene | | | | | | | | **No. of isolates from source** |
| --- | --- | --- | --- | --- | --- | --- | --- | --- | --- | --- | --- |
|  |  |  | **Ser22Gly A64G** | **Ala39Ser**  **G118T** | **Arg48Lys**  **G146A*** | **Thr85Ala**  **A256G** | **Thr86Ile**  **C257T** | **Ala122Ser**  **G364T*** | **Glu136Asp**  **G408T*** | **Val149Ile**  **G445A** |  |
| **CC1034**  (ST-6409) | 1 | 16 | - | - | - | - | ACA→ATA | - | - | - | 1(P) |
| **CC1287**  (ST-6414) | 1 | 4 |  |  |  |  |  |  | GAG→GAT |  | 1(WB) |
| **CC179** (ST:2209 ; 6421; 6424;  6426; 220;  4447) | 23 | 8-32 | - | - | - | - | ACA→ATA | - | GAG→GAT | - | 2(H) 9(WB) |
|  |  | 4-64 | - | - | - | - | - | - | GAG→GAT | - | 10(WB) |
|  |  | 2 | - | - | AGA→AAA | - |  | - | - | - | 1 (WB) |
|  |  | 8 | AGT→GGT | - | - | - | ACA→ATA | - | - | - | 1(WB) |
| **CC206**  (ST:227; 122**)** | 8 | 8-64 | - | - | - | - | ACA→ATA | - | - | - | 2 (P) 5(H) |
|  |  | 32 | AGT→GGT | - | - | - | ACA→ATA | - | - | - | 1 (H) |
| **CC21**  (ST:19; 21; 50; 376; 251; 1459; 1943; 6393; 6436; 7211) | 31 | 4-64 | - | - | - | - | ACA→ATA | - | - | - | 16(H) 7(P) |
|  |  | 128 | AGT→GGT | - | - | - | ACA→ATA | - | - | - | 2(H) 5(P) |
|  |  | 64 | - | - | - | - | ACA→ATA | - | GAG→GAT | - | 1(H) |
| **CC257**  (ST:257; 824) | 8 | 16 | - | - | - | - | ACA→ATA | - | - | - | 1(H) |
|  |  | 32-64 | AGT→GGT | - | - | - | ACA→ATA | - | - | - | 2(H) 1(P) |
|  |  | 16-64 | AGT→GGT | GCA→TCA | - | - | ACA→ATA | - | - | - | 4(H) |
| **CC283**  (ST: 6382; 7210) | 2 | 4-16 | - |  | - | - | ACA→ATA | - | - | - | 1(H) 1(P) |
| **CC353**  (ST:5; 353; 356; 3285) | 41 | 2 | AGT→GGT |  | - | - | - | - | - | - | 1(H) |
|  |  | 4-64 | - |  | - | - | ACA→ATA | - | - | - | 7(H) 4(P) |
|  |  | 8-128 | AGT→GGT |  | - | - | ACA→ATA | - | - | - | 19(H) 7(P) |
|  |  | 4 | - |  | - | - | ACA→ATA | - | - | GTT→ATT | 1(P) |
|  |  | 128 | AGT→GGT | GCA→TCA | - | - | ACA→ATA | - | - | - | 1(P) |
|  |  | 8 | AGT→GGT | - | - | - | ACA→ATA | - | - | GTT→ATT | 1(H) |
| **CC354**  (ST:354; 6466) | 8 | 32-64 | - | - | - | - | ACA→ATA | - | - | - | 1(H) 3(P) |
|  |  | 32-64 | AGT→GGT | - | - | - | ACA→ATA | - | - | - | 3(P) |
|  |  | 64 | - | - | - | - | ACA→ATA | - | GAG→GAT | - | 1(H) |
| **CC42**  (ST-42) | 3 | 64 | - | - | - | - | ACA→ATA | - | - | - | 1(P) |
|  |  | 8 | - | - | - | - | ACA→ATA | GCG→TCG | - | - | 1(P) |
|  |  | 1 | - | - | - | ACA→GCA | - | - | - | - | 1(H) |
| **CC443**  (ST:51; 6391; 7208) | 9 | 16-64 | - | - | - | - | ACA→ATA | - | - | - | 2(H) 5(P) |
|  |  | 16 | AGT→GGT |  | - | - | ACA→ATA | - | - | - | 1(H) |
|  |  | 32 | AGT→GGT | GCA→TCA | - | - | ACA→ATA | - | - | - | 1(H) |
| **CC446**  (ST:446; 6392) | 3 | 16-32 | - | - | - | - | ACA→ATA | - | - | - | 2(H) |
|  |  | 128 | AGT→GGT | - | - | - | ACA→ATA | - | GAG→GAT | - | 1(P) |
| **CC45**  (ST:45; 137; 233; 583; 2067) | 5 | 8 | - | - | - | ACA→GCA | ACA→ATA | - | - | - | 1(WB) |
|  |  | 8-64 | - | - | - | - | ACA→ATA | - | - | - | 1(H) 2 (P) |
|  |  | 16 | AGT→GGT | - | - | - | ACA→ATA | - | - | - | 1(P) |
| **CC460**  (ST:670; 6467) | 2 | 32 | AGT→GGT | - | - | - | ACA→ATA | - | - | - | 1(H) |
|  |  | 2 | AGT→GGT | - | - | - | - | - | - | - | 1(H) |
| **CC464**  (ST-464) | 15 | 8-64 | - | - | - | - | ACA→ATA | - | - | - | 1(H) 11(P) |
|  |  | 16-128 | AGT→GGT | - | - | - | ACA→ATA | - | - | - | 1(H) 2(P) |
| **CC48**  (ST:429; 475; 918) | 10 | 8-64 | - | - | - | - | ACA→ATA | - | - | - | 3(H) 2(P) |
|  |  | 16-64 | AGT→GGT | - | - | - | ACA→ATA | - | - | - | 4(H) 1(P) |
| **CC52**  (ST-2066) | 4 | 16 | - | - | - | - | ACA→ATA | - | - | - | 1(P) |
|  |  | 32-64 | AGT→GGT | - | - | - | ACA→ATA | - | - | - | 2(H) |
|  |  | 64 | AGT→GGT | - | AGA→AAA |  | ACA→ATA | - | GAG→GAT | - | 1(H) |
| **CC574**  (ST-305) | 2 | 16 | - | - | - | - | ACA→ATA | - | - | - | 1(H) |
|  |  | 32 | AGT→GGT | - | - | - | ACA→ATA | - | - | - | 1(H) |
| **CC607**  (ST-607) | 3 | 2-64 | AGT→GGT | - | - | - | ACA→ATA | - | - | - | 1(H) 2(P) |
| **CC658**  (ST:658; 6468) | 5 | 2 | AGT→GGT | - | - | - | - | - | - | - | 1(H) |
|  |  | 64 | - | - | - | - | ACA→ATA | - | - | - | 1(P) |
|  |  | 16-64 | AGT→GGT | - | - | - | ACA→ATA | - | - | - | 1(H) 2(P) |
| 692  (ST-692) | 1 | 4 | - | - | - | - | - | - | GAG→GAT | - | 1(WB) |
| **CC952**  (ST: 2110; 2111; 6228; 6397; 6399; 6402; 6408; 6434) | 10 | 8-16 | - | - | - | - | ACA→ATA | - | GAG→GAT | - | 2(WB) |
|  |  | 2-8 | - | - | - | - | - | - | GAG→GAT | - | 8(WB) |
| **CCNA** | 82 | 2 | AGT→GGT | - | - | - |  | - | - | - | 1(H) |
|  |  | 4-256 | - | - | - | - | ACA→ATA | - | - | - | 1(H)21(P) 6(WB) |
|  |  | 2-64 | - | - | - | - | - | - | GAG→GAT | - | 42(WB) |
|  |  | 16-128 | AGT→GGT | - | - | - | ACA→ATA | - | - | - | 2(H) 4(P) |
|  |  | 8-16 | - | - | - | - | ACA→ATA | - | GAG→GAT | - | 4(WB) |
|  |  | 8 | - | GCA→TCA | - | - | ACA→ATA | - | GAG→GAT | - | 1(WB) |

CC, clonal complex; MIC, minimum inhibitory concentration; CCNA, undefined clonal complex; QRDR, quinolone resistance-determining region, H, human; P, poultry product; WB, wild bird. Four ciprofloxacin resistant *C. jejuni* isolates from wild birds assigned to undefined clonal complex (ST-6430, ST-6400, ST-3755, ST-6431) had no point missense mutations in QRDR. *C. jejuni* isolates from human (n=4) and wild birds (n=7) was sensitive to ciprofloxacin and had no point mutation in QRDR. *****Novel missense mutation found in this study.
